# Robust Tipless Positioning Device for Near-Field Investigations: Press and Roll Scan (PROscan)

**DOI:** 10.1021/acsnano.2c05047

**Published:** 2022-08-03

**Authors:** Hsuan-Wei Liu, Michael A. Becker, Korenobu Matsuzaki, Randhir Kumar, Stephan Götzinger, Vahid Sandoghdar

**Affiliations:** † Max Planck Institute for the Science of Light, D-91058 Erlangen, Germany; ‡ Department of Physics, Friedrich-Alexander-Universität Erlangen-Nürnberg, D-91058 Erlangen, Germany; § Graduate School in Advanced Optical Technologies (SAOT), Friedrich-Alexander-Universität Erlangen-Nürnberg,, D-91052 Erlangen, Germany

**Keywords:** scanning probe microscopy, nano-optics, quantum dots, nanoparticle, fluorescence enhancement, near-field spectroscopy

## Abstract

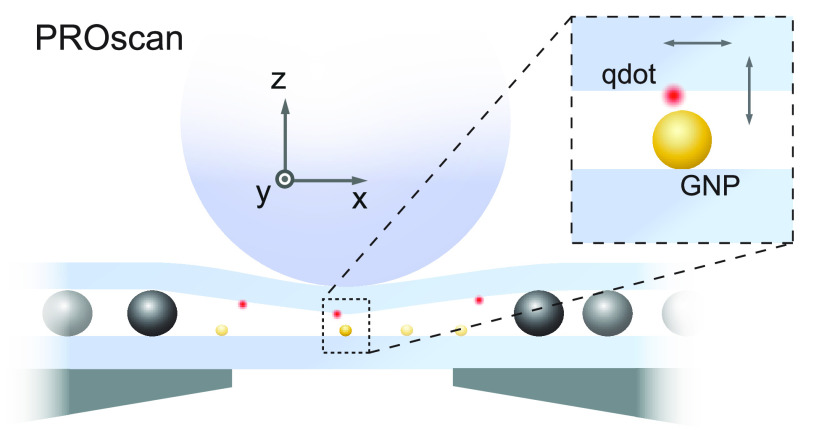

Scanning probe microscopes scan and manipulate a sharp tip in the immediate vicinity of a sample surface. The limited bandwidth of the feedback mechanism used for stabilizing the separation between the tip and the sample makes the fragile nanoscopic tip very susceptible to mechanical instabilities. We propose, demonstrate, and characterize an alternative device based on bulging a thin substrate against a second substrate and rolling them with respect to each other. We showcase the power of this method by placing gold nanoparticles and semiconductor quantum dots on the two opposite substrates and positioning them with nanometer precision to enhance the fluorescence intensity and emission rate. Furthermore, we exhibit the passive mechanical stability of the system over more than 1 h. Our design concept finds applications in a variety of other scientific and technological contexts, where nanoscopic features have to be positioned and kept near contact with each other.

## Introduction

Since the invention of the scanning tunneling microscope (STM) in 1981,^1^ scanning probe microscopy (SPM) has become indispensable in nanoscience and surface science, where structures below the optical diffraction limit are investigated down to individual atoms. Two of the most prominent SPM methods that followed STM are atomic force microscopy (AFM)^2^ and scanning near-field optical microscopy (SNOM).^3,4^ The central and common feature of all SPM methods is a sharp tip that is operated in the immediate vicinity of a sample surface.

In addition to high-resolution imaging, SPMs have also proven very valuable for manipulation and control of nanoscopic interactions. A leading example in nano-optical studies has been the placement of nanoantenna structures at the end of sharp dielectric tips so as to couple them to emitters in a controlled fashion.^5−12^ Similar experiments have also been reported on gap plasmons generated by coupling an emitter to the junction between a metallic tip and a metalized substrate.^13−17^ However, these efforts remain very challenging and often not accessible to the wider use.

Aside from the difficulties encountered in the fabrication, characterization and handling of tips, an important hurdle in SPM-based efforts is the low bandwidth of the feedback signal used to maintain the tip–sample separation at a nanoscopic value. This makes SPMs highly susceptible to mechanical perturbations and irreversible tip damage. With shear-force feedback control, which is commonly used in SNOM,^18^ it is particularly difficult to control distances with better than 1 nm precision. This has not been a limiting factor for common SNOM experiments with resolution between 10 and 100 nm; however, recent progress in plasmonics has shown that optimal enhancements take place at near-to-contact distances,^19^ leading to phenomena such as strong coupling.^20−22^ In this work, we present a tipless platform for performing mechanically robust and controllable experiments deep in the optical near-field. The general concept of the device, however, lends itself to a wide range of applications, where nano-objects and surfaces are coupled to each other locally at nanometer separations.

## Results and Discussion

### PROscan Concept and Its Realization Scheme

**Figure 1:**
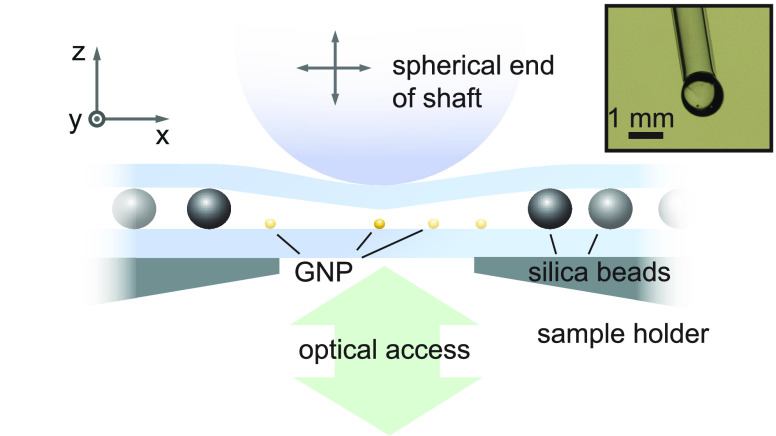
Schematic illustration of a PROscan device. A thin substrate is locally bulged through pressure and rolled against a second substrate. The sketch presents the example of an application in which gold nanoparticles (GNP) placed on the bottom substrate serve as nanoantennas, interacting with a medium prepared on the upper substrate. Inset: Glass capillary with a spherical end, melted by a CO_2_ laser, used as a handle to press and roll the top substrate.

Figure 1 sketches the heart of the device. The main strategy is to bulge a substrate by a very small amount toward a second substrate that is designed to be much less flexible. In our current work, we chose the flexible substrate to be a cover glass of thickness 100 μm, which was thoroughly cleaned with deionized water and nonhalogenated solvents (acetone and isopropanol). We used a conventional microscope cover glass of thickness 170 μm as the lower substrate and supported it with a steel bracket that contained a central opening of 5 mm to allow for optical detection through an oil-immersion microscope objective. The substrate was treated with oxygen plasma to remove organic residuals and to make its surface hydrophilic.

To produce a miniaturized handle for exerting local pressure on the upper substrate, we melted the end of a glass capillary to a quasi-spherical shape of diameter ∼1 mm (see inset in Figure 1). A piezoelectric element (Piezosystem Jena, Tritor 38) was used to position the capillary shaft. A small amount of epoxy was applied to the spherical end to enhance surface friction. As we shall see below, a typical voltage of a few tens of volts is sufficient to bend the upper cover glass by several 100 nm over a lateral span of about 10 mm.

**Figure 2:**
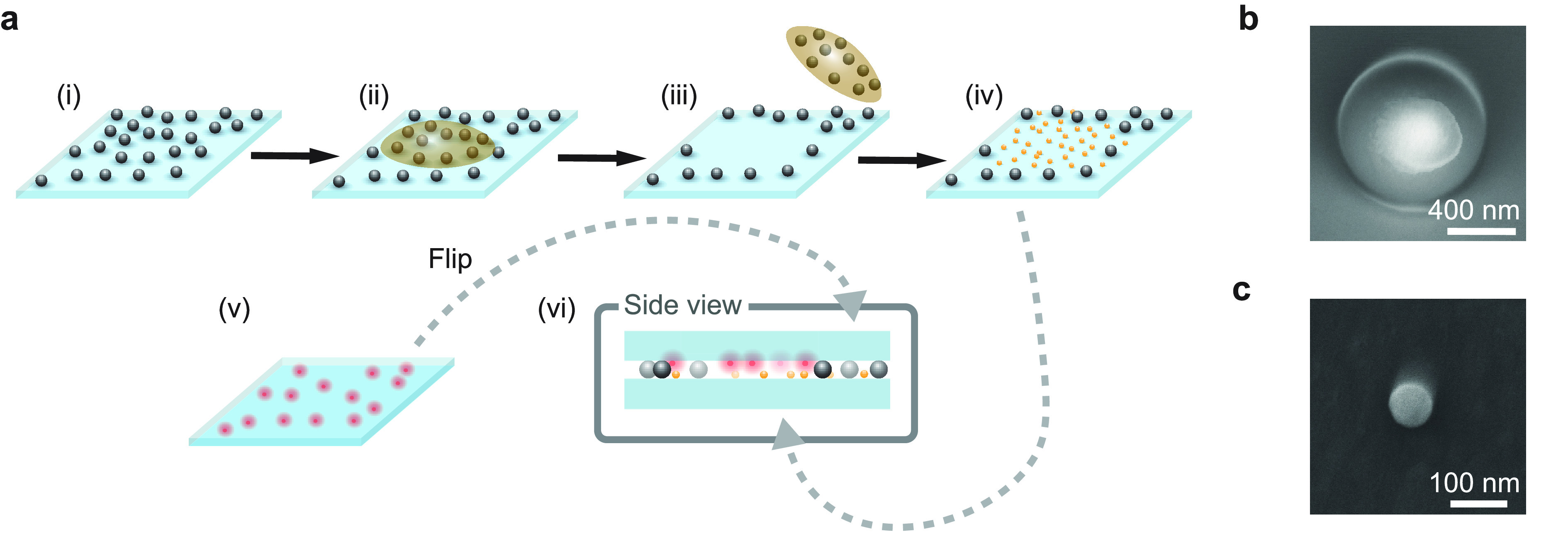
(a) Sample preparation procedure: Silica spacer beads are spin-coated onto a clean (bottom) glass substrate (i). A cleaning polymer is applied to the central region (ii) and is removed after drying (iii). Nanoprobes are placed onto the same substrate (iv). Emitters such as molecules or quantum dots are placed onto a second (top) substrate (v). The top substrate is flipped and placed on the bottom substrate (vi). (b) Scanning electron micrograph of a single silica bead with a diameter of 800 nm. (c) Helium-ion microscope image of an individual GNP.

By placing spherical spacers between the two substrates, we adjust their separation to the desired range and allow for rolling the entire upper substrate. Figure 2a illustrates the fabrication process. We first spin-coated silica beads (Bangs Laboratories Inc.) on the lower substrates (30 s at 3000 rpm) to yield an average coverage of one particle per 100 × 100 μm. In our current report, we varied the silica bead diameter between 0.8 and 3 μm. Figure 2b presents an electron microscope (EM) micrograph of such a bead. The particles are then removed from the central region of the substrate by an adhesive cleaning polymer (First Contact, Photonic Cleaning Technologies). Here, we first apply a solution of 100 μL of the cleaning polymer to the center of the bottom substrate to form a droplet spread over roughly 5–10 mm. After being dried, the polymer is simply peeled off to remove the silica beads that were in contact with it (see step (iii) in Figure 2a). This process leaves the surface in a very clean state. Nevertheless, we expose the assembly to 5 min of O_2_ plasma in order to eliminate any nanoscopic residual amount of polymer. The clean central area allows one to press the sample in the axial direction until the two glass substrates touch.

To mimic the sharp end of an SPM tip, one of the substrates is decorated with a well-defined “nanoprobe” of choice. In our current work, we demonstrate the principle of this step with gold nanoparticles (GNPs) with a diameter of 80 nm, which we spin-coat on the lower substrate (30 s at 3000 rpm; see step (iv) in Figure 2a). Figure 2c shows a helium-ion microscope image of a GNP. We chose a GNP coverage corresponding to an average particle separation of 10 μm to facilitate diffraction-limited optical detection in an uncrowded region.

The medium of interest to be studied, e.g., thin films, nanoparticles, or molecules, is placed on the substrate opposite to the one containing the nanoprobe. In this work, we used CdSe/CdS core/shell colloidal quantum dots (qdot) with a core size of 4 nm and a total diameter of 16 nm.^10,23^ We sparsely deposited the qdots onto the upper glass substrate. To do this, a toluene solution of the colloidal qdots was diluted to nanomolar concentrations. Twenty microliters of this suspension was spin-coated in a two-step process (30 s at 1000 rpm followed by 3 s at 3000 rpm). Subsequently, the upper substrate was flipped and placed on top of the bottom substrate (see step (vi) in Figure 2a). Various modes of imaging (dark-field, iSCAT, fluorescence, etc.) can be employed to identify the individual nanoprobes.

As we demonstrate below, the upper substrate can be pressed and rolled to scan a qdot against a GNP with nanometer precision. We thus refer to this technique as PROscan. We emphasize that the choices of the two substrates (e.g., material, thickness), rolling mechanism (e.g., choice of beads, nanolubrication), and the nanoprobe can be varied and optimized for different applications. In particular, one could use bottom-up (e.g., self-assembly) or top-down (e.g., electron beam lithography) fabrication for the realization of various nanoprobes such as cones.^10,24^

#### Lateral Scan and Position Control

**Figure 3:**
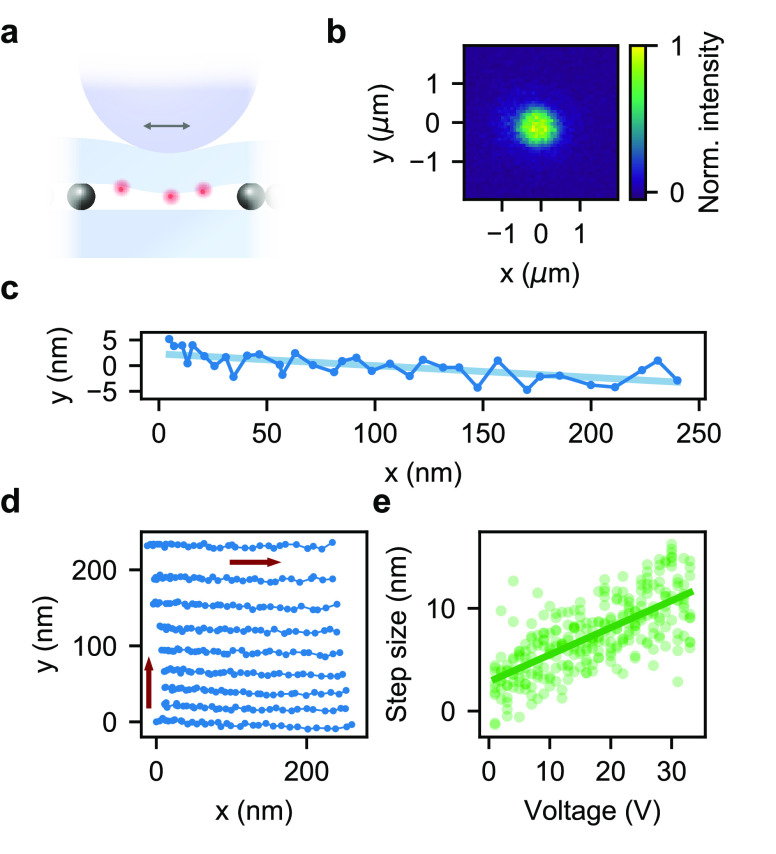
(a) Schematics of the measurement configuration used for characterizing lateral scans. Red points on the upper substrate signify individual qdots dispersed on the top substrate. (b) Fluorescence image of an individual qdot. Localization of the qdot in images recorded at each location during the rolling and scanning process maps the displacement of the top substrate. (c) Exemplary trajectory of a qdot during an *x* scan of the top substrate. The line depicts a linear fit to the data. (d) Example of the qdot trajectories recorded during a 2D scan of the top substrate. The directions of the *x* and *y* scans are indicated by the red arrows. (e) Variations of the measured step size in *x* direction after the turning points of the 2D scan for all lateral forward scans shown in (d). Solid line represents a linear fit to the data. Our measurements were performed in a temperature-controlled environment that is stable to better than 0.5 °C.

PROscan allows one to approach a selected nanoprobe both laterally and axially to the location of the sample under investigation. A range of signals can be used to monitor or control the separation of the two substrates during this process. In this work, we used a combination of interferometry, modification of the GNP plasmon resonance, and fluorescence enhancement of qdots. To examine the quality of the lateral position control, we recorded the trajectories of single qdots while rolling the upper substrate against a naked lower substrate (see Figure 3a). A fluorescence image was recorded at each lateral *xy* scan, and the point-spread function (PSF) of the qdot (see Figure 3b) was analyzed by fitting a two-dimensional Gaussian function. The high signal-to-noise ratio (SNR) allowed us to localize a qdot^25^ with an average precision of 2.4 nm in both directions.

Figure 3c displays an example of a trajectory, while a linear voltage ramp of 1 V per step was applied to the piezoelement along the *x* direction without the use of any feedback control. A linear fit to the data yields a slope of *m* = −0.023 ± 0.005, corresponding to a tilt angle of 1.3 ± 0.3°. In Figure 3d, we present a series of *x* scans recorded at different *y* locations. Here, we observe an average tilt angle of 1.5° with a standard variation of 0.5°. Such a small tilt could be caused by a slight misalignment of the piezoelement with respect to the camera, but the variations among the individual scans lead us to attribute the observed variation to a small cross talk between the *x* and *y* axes. Moreover, we note that the step size increases at higher applied voltages while it is smaller again after the turning points in a zigzag scan scheme. However, the step size reaches a steady state if one scans only in one direction (see Figure 6d). Figure 3e displays the measured step sizes as a function of the applied voltage. We find an average step size of 7.3 nm with a standard deviation of 3.5 nm in the scan direction and a lateral jitter of 2.7 nm.

In the measurements presented in this work, we scanned at 0.5–10 steps per second, corresponding to traveled distances ranging from less than 1 nm to several micrometers per step. This slow speed was dictated by the integration time that was necessary for recording the optical signal. However, we have verified that we can reach scanning speeds in the order of 1 μm/ms under the typical load used here. We note that the mechanical scanning speed of PROscan is ultimately limited by the rheological properties of the substrates and spacer spheres. A quantitative characterization and optimization of these phenomena goes beyond the scope of our current article.

In conventional SPM, the lateral position of the tip with respect to the sample is usually passively scanned by piezoelectric elements although actively stabilized scanners have also become common. The finesse of the scanning grid depends on the application and can be as small as angstroms. The same instrumentation can also be used for applications where the probe is used to manipulate the sample, e.g., in AFM lithography^26,27^ or plasmonic nanoantennas.^5−17^ In these cases, a passive predetermined knowledge of the tip–sample position is not a requirement, and it would be sufficient to measure and monitor the relative position during the experiment. The data in Figure 3c,d show that although the lateral scans in this very simple implementation of PROscan are not as uniform as in conventional SPM, they do achieve nanometer precision. This performance can be further improved by employing additional feedback mechanisms, e.g., by measuring the actual position of an emitter and correcting for small step size errors in an iterative manner.

#### Axial Position Control

The most crucial step in operating an SPM is to approach a nanoprobe to a sample with nanometer or sub-nanometer precision. In practice, one usually reduces the distance with a translation stage carefully until the characteristic near-field signal is detected. In STM, one uses the tunneling current as a measure for the sample-probe distance, whereas the shear-force signal is used in SNOM.^18,28^ In PROscan, one can use optical measurements to determine and monitor the position of the upper substrate with nanometer precision. In the following, we present two methods to accurately measure distances between the substrates without the need for any feedback mechanism.

**Figure 4:**
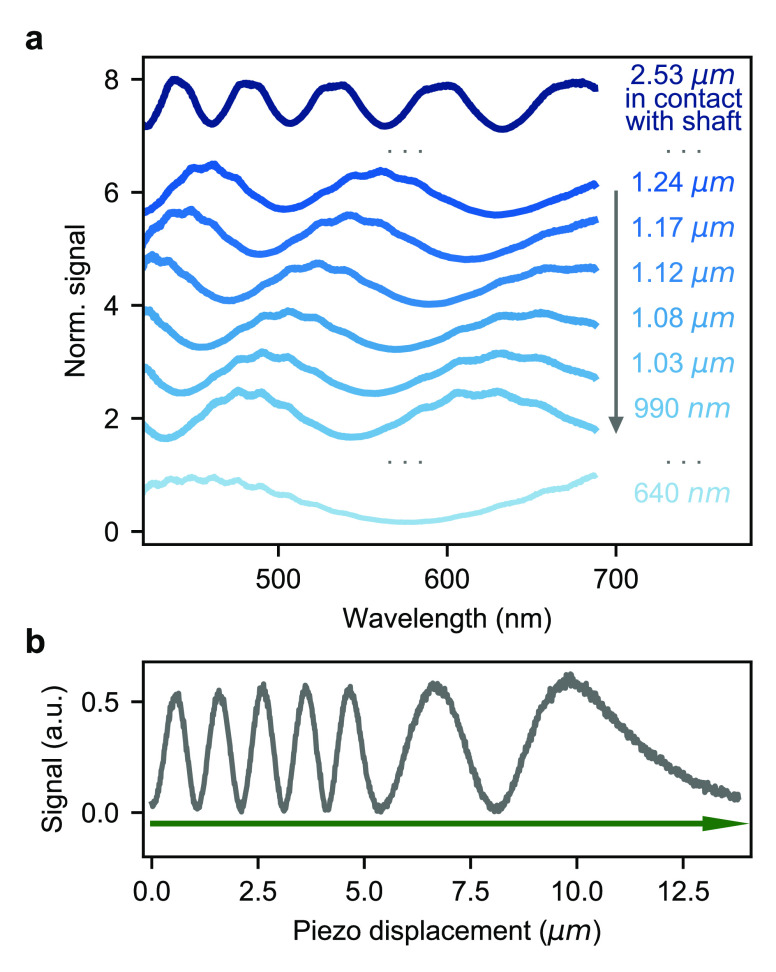
(a) Interference spectra for different gap sizes between the two glass substrates using white-light illumination. The dark blue spectrum on top was recorded as the shaft contacted the top substrate. (b) Reflected intensity of a laser beam at λ = 532 nm as the upper substrate is lowered further. A piezo displacement of about 13 μm leads to a substrate displacement of about 1.9 μm.

For gaps larger than the wavelength of visible light, interference fringes can be used to deduce the distance between the two substrates, which form a Fabry–Perot interferometer. As shown in Figure 4a, we use a white-light excitation source to record the Fabry–Perot spectrum at each axial position. Analysis of this information allows us to deduce an absolute distance *d* between the two substrates according to the formula  obtained from the definition of the free spectral range for an optical cavity formed in air. Here, λ_1_ and λ_2_ denote the wavelengths of two neighboring Fabry–Perot resonances. This simple strategy ceases to work at small separations, where one no longer records full oscillations (see lower trace in Figure 4a).

Hence, we augment our knowledge of the substrate separation by measuring the relative displacement using monochromatic interferometry (λ = 532 nm). As shown in Figure 4b, each oscillation indicates a displacement of λ/2 = 266 nm. The monotonic change in the periodicity as a function of the applied piezovoltage indicates the reduction in the bending capability of the substrate. For this measurement we used 3 μm sized silica beads in order to start with a larger separation between the substrates.

The interferometric measurements discussed above cannot report on the separation between a nanoscopic probe such as a GNP and the substrate on the opposite side. To demonstrate that we can control this distance with nanometer precision in the near-field, we measured the modification of the plasmon resonance of a single GNP^29,30^ placed on the bottom substrate, while lowering the upper glass substrate.

As we press on the PROscan device (see Figure 1), the central opening in the sample holder also allows for a slight bend of the lower substrate. This overall displacement buffers the relative motion of the two substrates so that a larger motion of the pressing shaft is needed to actuate a small shortening of the distance between the GNP and the upper substrate. As a result, it can happen that the vertical piezoelement reaches its maximum range before achieving the desired gap. In this case, we first reduce the applied voltage, and then manually lower the top substrate with a micrometer stage until we regain the same axial position, whereby we use the PSF of the GNP on the lower substrate to regain the same focus quality in the optical image. This procedure is repeated iteratively until the desired gap between the upper and lower substrates is reached. Overall, this bending phenomenon allows one to reduce the gap more smoothly and acts to magnify the piezostep resolution. We estimate a maximum error of 5 nm in the stitching procedure.

**Figure 5:**
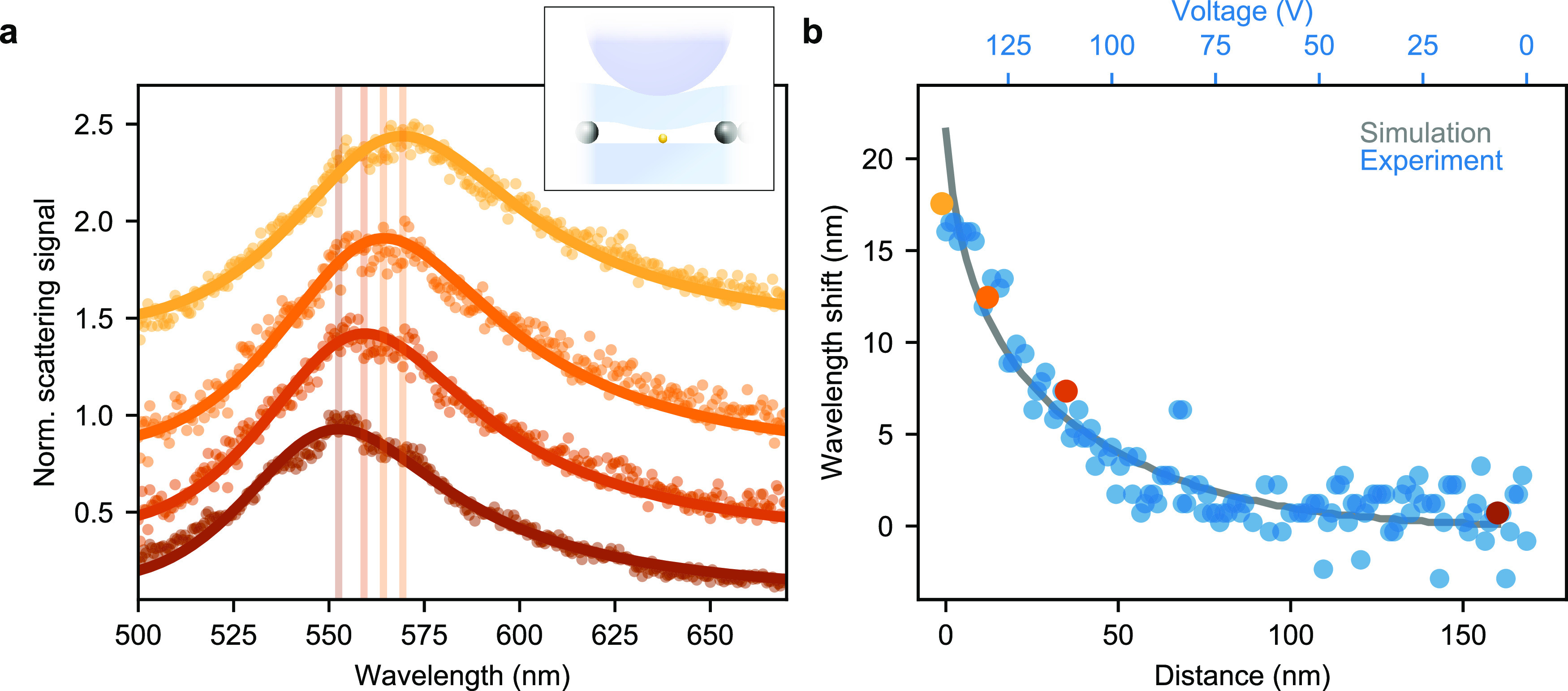
(a) Normalized scattering spectra of an individual GNP on a glass substrate recorded in a dark-field arrangement for four different axial positions as marked by the correspondingly color-coded scatter points in (b). Vertical lines indicate the maximum of the fit function. Inset: Measurement configuration. (b) Measured wavelength shifts (symbols) extracted from plasmon spectra recorded at different axial positions as a function of the applied effective piezovoltage (upper horizontal axis). Gray solid curve represents the theoretical resonance shift simulated for 81 different gap distances (lower horizontal axis) to which the experimental data are fitted. Color-coded symbols indicate the four data points corresponding to the spectra shown in (a).

In Figure 5a, we present four examples of plasmon spectra recorded at different separations of the upper substrate. To determine the resonance wavelength, we fit the normalized experimental spectrum with its theoretical counterpart based on the dipolar approximation and taking into account an effective polarizability for the GNP.^30,31^ The spectra clearly report on the expected near-field change in the resonance frequency,^29,30^ which can be intuitively understood as the consequence of the interaction between the dipole moment associated with the plasmon mode and its mirror image in the glass substrate.

The symbols in Figure 5b plot the extracted plasmon resonance as a function of the effective applied piezovoltage in the *z* direction (upper horizontal axis). The color-coded data points indicate the corresponding spectra in Figure 5a. The gray curve also presents the calculated resonance shifts resulting from finite-difference time domain simulations performed at 81 different distances (lower horizontal axis). To establish a common horizontal axis between the experimental and simulated data, we fit the former with the interpolated simulation results while allowing for a linear scaling between the applied voltage and the achieved displacement. This yields a conversion rate of −1.2 ± 0.2 nm/V, which is considerably lower than the value of −380 nm/V expected for the case without load. In other words, the precision in changing the gap between the substrates is increased or, equivalently, the noise is dampend. Indeed, Figure 5b displays a good agreement in the predicted trend of the wavelength shift as a function of the GNP–substrate separation, indicating that we achieve nanometer control of the axial position.

### Controlled Enhancement of Fluorescence from a Single Quantum Dot Coupled to a Gold Nanoparticle

Modification of fluorescence in the near-field of plasmonic nanostructures has continuously fascinated scientists since the early 1980s.^32−34^ A controlled and routine realization of this simple-seeming idea, however, continues to be elusive because it requires (1) a high degree of control in the shape, size, and material of the metallic nanostructure, (2) nanometer precision in placement of the nanostructure with respect to an emitter, (3) good control of the orientation of the emitter’s dipole with respect to the nanostructure, and (4) well-defined polarization of the excitation and illumination optical fields.^9^ Various attempts have addressed these issues using statistical strategies.^20−22,35^ Nearly two decades ago, we introduced a simple idea for performing controlled single-emitter studies: a gold nanoparticle was placed at the end of a glass tip to act as a nanoantenna, which could be positioned in all three dimensions with nanometer precision using the machinery of a SNOM device.^5,7,8^ While the quantitative control in this approach has attracted some attention,^10−12,16^ its widespread use has been hampered by the experimental complexity that accompanies single-emitter SPM studies. We now demonstrate that PROscan can achieve comparable results in a more robust arrangement. We present a concrete example, where a single qdot is coupled to a plasmonic nanoparticle.

**Figure 6:**
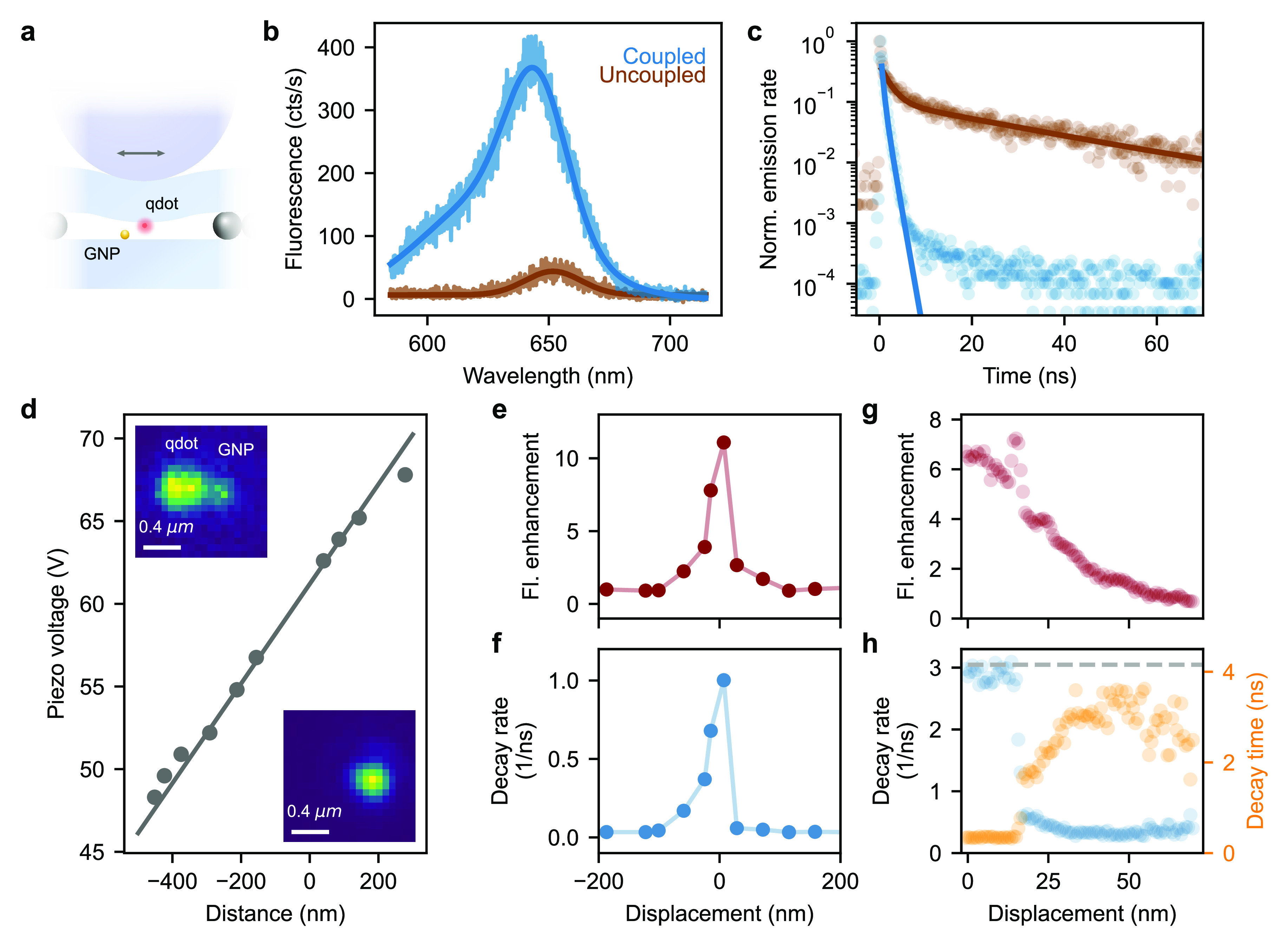
(a) Schematics of the measurement configuration: A single CdSe/CdS qdot is laterally scanned over an individual GNP. (b) Fluorescence spectra of the qdot before (brown) and after coupling (blue) to the GNP. (c) Fluorescence decay time measurement of the uncoupled (brown) and coupled (blue) qdot. Both curves are fitted with a biexponential decay function (solid lines). The part of the data at longer times was not taken into account for the coupled case since it corresponds to a signal that is 3 orders of magnitude weaker. (d) Applied piezovoltage as a function of the lateral GNP–qdot distance extracted from 2D localization fits to fluorescence images of the qdot. A linear fit is depicted as a gray solid line. Top inset: Fluorescence image of a qdot displaced from the GNP. The latter also gives rise to very weak fluorescence. Bottom inset: Fluorescence image of the coupled qdot–GNP system. (e) Measured fluorescence signal as a function of the lateral displacement during a coarse scan of the qdot across the GNP. (f) Fluorescence decay rate (inverse of the fluorescence lifetime) recorded simultaneously as (e) for each step of the coarse scan. (g) Similar measurement as in (e) with a finer sub-nanometer step size. (h) Fluorescence decay rate (blue) and decay time (orange) as a function of the displacement. The gray dashed line represents the measurement decay rate limit dictated by the instrument response function.

As depicted in Figure 6a, we placed qdots on one substrate and GNPs on a second substrate. For pulsed excitation well below saturation, the change in the fluorescence intensity is mainly expressed as the product of the modification factors in the excitation rate, quantum efficiency and collection efficiency.^36^ In our case, the latter is not a decisive factor since we collect the emitted light very efficiently with a high-NA microscope objective.

In Figure 6b, we show the emission spectra of a single qdot before (brown, center wavelength ∼652 nm) and after coupling to a GNP antenna (blue, center wavelength ∼643 nm), revealing an 11-fold fluorescence enhancement. We note a blue-shifted shoulder in the emission spectrum, which can be attributed to a charged exciton or trion emission.^23^ Furthermore, we observe a coupling-induced blue shift in the main emission peak. We attribute this effect to the fact that the GNP plasmon resonance peaks at 560 nm, which is considerably blue-shifted with respect to the emission spectrum of the qdot, thus more strongly enhancing its high-energy part.

We also recorded the fluorescence decay curve of the same qdot with and without the presence of the GNP. The results are shown by the symbols in Figure 6c. The photophysics of the nonblinking qdots used in our work^23^ allows for efficient access to biexciton emission, which can also be enhanced by a plasmonic antenna.^10,37,38^ As previously shown,^10^ the decay curve of the uncoupled qdot displays a clear biexponential behavior, with fast (τ_1_) and slow (τ_2_) components, corresponding to the biexciton and exciton decay processes, respectively. Fitting the rapid decay of the fluorescence signal over 3 orders of magnitude (see blue symbols in Figure 6c) yields a biexponential function. We find that τ_1_ is reduced from 2.1 ± 0.1 to 0.4 ± 0.1 ns, and τ_2_ is shortened from 29.4 ± 0.8 to 1.0 ± 0.1 ns.

The two measurements in Figure 6c were recorded as a part of a line scan, in which we moved a qdot across the GNP. The inset in the upper left corner of Figure 6d displays a fluorescence image of the qdot and the GNP (gold nanoparticles typically have a weak fluorescence signal^39^) at a large separation of about 400 nm. We extract the positions of the GNP and the qdot by fitting 2D Gaussian functions to their respective images. In this fashion, we deduce the distance between the qdot and the stationary GNP for each frame and plot it in Figure 6d against the applied piezovoltage. We note that a lack of the knowledge of the dipole orientation combined with the position dependence of the antenna effect on its radiation pattern prevents one from an accurate localization of the qdot in the near-field of the GNP (see lower right inset of Figure 6d). We, thus, only consider events before and after a considerable plasmonic coupling. Figure 6d shows that a linear function fits the data very well, confirming the findings discussed in Figure 3 and yielding a local displacement-to-voltage rate of 33.2 nm/V. Using this information, in Figure 6e, we show the normalized (to the fluorescence of the uncoupled qdot) integrated fluorescence signal of the qdot as it is scanned across the GNP. Furthermore, we plot the measured slow decay rate (exciton) in Figure 6f for each step. We observe an initial increase from 0.03 ns^–1^ for the uncoupled to 1 ns^–1^ for the maximally coupled qdot in this scan. The asymmetric line profile might be caused by the tilt of the qdot dipole moment.^9,36^

To examine the near-field interaction more closely, we repeated the measurements with a finer spatial resolution. Therefore, we again approached the qdot to the GNP until we observed a change of the fluorescence intensity at an approximate separation of ∼70 nm. Then, we laterally reduced the distance with a finer step size and recorded the fluorescence enhancement and temporal decay as presented in Figure 6g,h, respectively. Acquiring independent information about the step size is now more challenging because the step size per applied unit of voltage is not the same for different scan conditions such that we cannot use the calibration obtained from Figure 6d. To estimate the scanned distance, we thus set the full width at half-maximum of the measured fluorescence signal of the fine scan in Figure 6g equal to that measured for the coarse scan presented in Figure 6d.

The smooth profile of the fluorescence signal in Figure 6g demonstrates the ability of PROscan to explore the near-field of a gold nanoantenna with nanometer precision. For example, one notices a very sharp feature at about *x* = 15 nm. Given its well-defined rise and fall over a period longer than 10 s (integration time per point was about 2 s), we believe this event is not an artifact and attribute it to the presence of a local sharp protrusion in the GNP. Indeed, the fluorescence lifetime (right axis) and fluorescence decay rate (left axis) also undergo a rapid change at *x* = 15 nm (see Figure 6h). The lifetime starts at about 3 ns and falls, first softly and then very quickly, as the qdots approach the GNP. The flat region at smaller distances denotes the finite instrument response time of our detector marked by the dashed gray line. Such a rapid shortening of the fluorescence lifetime is expected and is mostly due to the onset of nonradiative decay channels very close to gold.^9,36^

We note that the fluorescence signal in Figure 6e,g continues to increase as the qdot becomes closer to the GNP; that is, we do not see a clear sign of quenching. This is because the plasmon resonance of the GNP has a strong overlap with the broad absorption band of the qdot. Thus, the local excitation strength continues to grow as the qdot and GNP become closer. The competition between quenching and excitation enhancement is further complicated by their dependence on the dipole orientation.

### Stability Studies

A decisive advantage of PROscan in comparison with conventional SPM platforms is the ability to engage in deep near-field interactions with high mechanical stability although the quantitative details of the system depend on the specifics of the choices of the substrates and holders.^40^ We now show that the two substrates under the load of the capillary shaft behave as a monolithic rigid system, reducing sensitivity to external vibrations.

**Figure 7:**
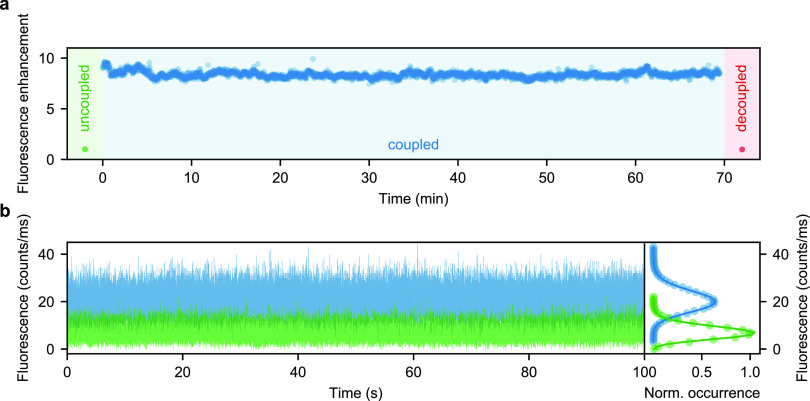
(a) Fluorescence enhancement of the single qdot coupled to a single gold nanoparticle over the course of 70 min (blue). The green and red data points depict the fluorescence enhancement before and after the coupling event, respectively. (b) Left: Collected emission from an uncoupled (green) and coupled (blue) qdot presented in 1 ms bins over the course of 100 s. Right: Histograms of the qdot fluorescence intensity in the coupled and uncoupled states. Solid curves show that the histograms fit Poisson distributions very well.

To demonstrate this feature, we monitored the fluorescence spectra of single qdots coupled to individual GNPs over more than 1 h. Fluorescence enhancement is a particularly good measure for the system’s stability because nanometer displacements between the nanoantenna and the qdot can drastically alter the signal. In Figure 7a, we showcase the stability of the PROscan device. We attribute small fluctuations of the measured intensity to charging events of the qdot. To verify that both the qdot and the GNP are still placed on separate substrates during the long observation time (i.e., we did not accidentally pick one up), we disengaged the upper substrate and confirmed that the qdot fluorescence was reduced to its uncoupled value. The green and red data points in Figure 7a mark the fluorescence of the qdot before coupling and after decoupling, respectively.

In Figure 7b, we also plot the emitted fluorescence of a coupled qdot–GNP system (blue) recorded with higher temporal resolution on an avalanche photodiode. We observe that the emitted intensity binned in one millisecond time intervals follows a Poisson distribution, similar to the emission trace of the same uncoupled nonblinking qdot (green). Hence, we conclude that there were no vibrations or oscillations that could have affected the distribution.

## Conclusions

Over the years, there have been many reports on the realization of measurement setups and geometries with inherent stability against mechanical vibrations, e.g., to investigate electrical and thermal conductivity of single molecules and atoms.^41−46^ These structures reach sub-nanometer precision in one dimension, but they have not demonstrated lateral scanning and positioning of a nanoprobe against a sample. We have shown that PROscan provides a sufficiently fine scannability to map the near-field coupling of a semiconductor qdot to a gold nanoantenna. We reported a substantial fluorescence enhancement during a horizontal scan accompanied by a reduction of the measured decay time. In particular, we used this coupled system to demonstrate the high mechanical stability of this passive device over a period longer than 1 h.

The PROscan apparatus is a powerful and yet simple alternative to conventional tip-based scanning probe techniques for executing a variety of nanoscopic optical, electrical and thermoelectrical measurements with nanometer resolution and without a feedback mechanism. The tipless design can also be used for incorporating planar scanning devices,^47^ scanning superconducting interference devices,^48^ scanning electron transistors,^49^ and nitrogen vacancy centers in diamond.^50^ The performance of PROscan can be improved further by optimizing the material and thickness of the flexible substrate, the choice of the spacer beads, and adjusting the surface roughness and rheological properties of the two substrates, e.g, via suitable surface functionalization. These measures could accommodate finer and faster scans as well as very small nanoprobes below 10 nm.

## Methods

### Optical Measurements

In all fluorescence measurements, the qdots were excited in a wide-field arrangement with either a continuous-wave or pulsed (repetition rate of 4.1 MHz) laser beam at a wavelength of 532 nm focused on the back focal plane of the immersion-oil microscope objective (Olympus UPlanSApo 100×, NA = 1.4) in total internal reflection (TIR) geometry. The fluorescence was collected with the same microscope objective and spectrally filtered using a 550 nm long-pass filter. To select the fluorescence of single emitters spatially, a variable pinhole was introduced in a conjugate plane of the image plane using a telecentric lens system. For dark-field scattering measurements of individual GNPs, p-polarized white light (Energetiq EQ-99 LDLS) was focused into the back focal plane of the microscope objective, also in a TIR configuration. In this manner, a plasmon mode that is polarized orthogonal to the glass substrate was excited. The scattered light from the particle was collected by the same objective, and the reflected light was blocked in the Fourier plane by the edge of a razor blade. Here, one needs to account for changes of both the excitation spectrum and the scattering background caused by the bending of the substrates and by spurious interference effects. Both the fluorescence emission and the scattered light were sent to a nonpolarizing 70:30 beam splitter, where 30% of the light was sent to a fiber-coupled Czerny–Turner spectrometer (Andor Shamrock 303i or Kymera 328i) equipped with an EMCCD camera (Andor Newton). The remaining light was sent to another nonpolarizing 50:50 beam splitter. Here, half of the light was sent to an imaging camera (Hamamatsu ORCA-Flash 4.0), and for time-resolved measurements, the other half was sent to a Hanbury Brown–Twiss setup consisting of two single-photon avalanche photodiodes (PD-050-CTB, Micro Photon Devices). The signals from the single photon detectors and from the laser sync were sent to a time-correlated single-photon counting module (Time Tagger Ultra, Swabian Instruments). For interference measurements, the white-light source and the 532 nm laser were focused at the center of the back focal plane of the oil-immersion microscope objective to obtain a wide-field illumination under normal incidence.

### Simulations

In three-dimensional finite-difference time domain simulations (Lumerical Inc., Ansys) a total-field scattered-field source was used to calculate the scattering cross section of individual GNPs at a single frequency. To mimic the experimental conditions, the incident angle was set to 65° and the diameter of the GNP was set to 80 nm. The refractive index of the glass substrate was set to 1.5 and the simulation region was surrounded by a perfectly matched layer. The finest mesh size was set to 1 nm to achieve sufficient simulation accuracy. To extract the wavelength of the maximum scattering cross section, we simulated the scattering cross section in the range between 500–600 nm in steps of 5 nm. Next, we fit the simulated spectra with the calculated plasmon spectrum based on the scattering cross section as discussed in the text and obtain its maximum from the fit.

## Notes

The data that support the findings of this study are available from the corresponding author upon request and can be found on Zenodo.org (10.5281/zenodo.6393239).
